# Social and Structural Factors Associated with HIV Infection among Female Sex Workers Who Inject Drugs in the Mexico-US Border Region

**DOI:** 10.1371/journal.pone.0019048

**Published:** 2011-04-25

**Authors:** Steffanie A. Strathdee, Remedios Lozada, Gustavo Martinez, Alicia Vera, Melanie Rusch, Lucie Nguyen, Robin A. Pollini, Felipe Uribe-Salas, Leo Beletsky, Thomas L. Patterson

**Affiliations:** 1 Departments of Medicine and Psychiatry, School of Medicine, University of California San Diego, La Jolla, California, United States of America; 2 Prevencasa, A.C., Baja California, Mexico; 3 Salud y Desarollo Comunitario de Ciudad Juárez A.C. (SADEC) and Federación Méxicana de Asociaciones Privadas (FEMAP), Ciudad Juárez, Mexico; 4 El Colegio de la Frontera Norte, Tijuana, Mexico; University of Cape Town, South Africa

## Abstract

**Background:**

FSWs who inject drugs (FSW-IDUs) can acquire HIV through high risk sexual and injection behaviors. We studied correlates of HIV infection among FSW-IDUs in northern Mexico, where sex work is quasi-legal and syringes can be legally obtained without a prescription.

**Methods:**

FSW-IDUs>18 years old who reported injecting drugs and recent unprotected sex with clients in Tijuana and Ciudad Juarez underwent surveys and HIV/STI testing. Logistic regression identified correlates of HIV infection.

**Results:**

Of 620 FSW-IDUs, prevalence of HIV, gonorrhea, Chlamydia, trichomonas, syphilis titers ≥1∶8, or any of these infections was 5.3%, 4%, 13%, 35%, 10% and 72%, respectively. Compared to other FSW-IDUs, HIV-positive women were more likely to: have syphilis titers ≥1∶8 (36% vs. 9%, p<0.001), often/always inject drugs with clients (55% vs. 32%, p = 0.01), and experience confiscation of syringes by police (49% vs. 28%, p = 0.02). Factors independently associated with HIV infection were syphilis titers ≥1∶8, often/always injecting with clients and police confiscation of syringes. Women who obtained syringes from NEPs (needle exchange programs) within the last month had lower odds of HIV infection associated with active syphilis, but among non-NEP attenders, the odds of HIV infection associated with active syphilis was significantly elevated.

**Conclusions:**

Factors operating in both the micro-social environment (i.e., injecting drugs with clients) and policy environment (i.e., having syringes confiscated by police, attending NEPs) predominated as factors associated with risk of HIV infection, rather than individual-level risk behaviors. Interventions should target unjustified policing practices, clients' risk behaviors and HIV/STI prevention through NEPs.

## Introduction

Transmission of HIV/STIs from higher to lower risk populations may be potentiated in settings where there is a high proportion of FSWs who inject drugs (FSW-IDUs) because they can acquire and transmit HIV through two transmission routes: unprotected sex and injecting with contaminated syringes. FSW-IDUs are highly vulnerable to violence from clients, managers, intimate partners, injection partners and police, which may lead to a reduced capacity to control their working conditions and lower self-efficacy for negotiating condom use and refusing needle sharing [1]–[3]. Compared to other FSWs, FSW-IDUs may also be more likely to acquiesce to clients' demands for unprotected sex if they are suffering from drug-related withdrawal [4], [5]. IDUs who are highly visible, such as street-based IDUs who engage in survival sex may experience frequent police harassment leading them to rush injections, inject in shooting galleries, and avoid needle exchange programs (NEPs) [6]–[11].

Although estimates vary widely by region, overlap between FSW and IDU populations can be considerable. Studies of FSWs have reported the prevalence of injection drug use to range from 32% in Hanoi, Vietnam [12], 58% in Yunnan, China [13], 56% and 65% in Vancouver and Montreal, Canada, respectively [14], [15], 51% among seven cities in the United States [16], 71% in Glasgow, Scotland [17] and 82% in Amsterdam, the Netherlands [18]. In northern Mexican cities situated along the U.S. border, injection drug use was reported among 14% of FSWs in Ciudad Juarez (adjacent to El Paso, TX) and 22% in Tijuana (adjacent to San Diego, CA), but these proportions were likely underestimates. In a study conducted in these cities from 2004–2006, HIV prevalence among FSW-IDUs was twice as high as that of other FSWs (12% vs. 6%, respectively [4]), but the small number of FSW-IDUs in that study precluded an examination of HIV correlates.

Many countries uphold laws prohibiting prostitution, but in Mexico, sex work is quasi-legal. Tijuana and Ciudad Juarez, and other cities situated along Mexico's northern border with the United States attract male clients from both countries who seek out FSWs in *zona rojas* (red light districts), where sex work is tolerated. For example, in Tijuana a government regulation system operates whereby sex work permits are provided to FSWs aged 18 and over who may work in the city's Zona Roja (red light district). Women are required to undergo HIV and STI on a quarterly basis. However, it is estimated that half of FSWs work without permits. In Ciudad Juarez, no permit system for FSWs exists, but sex work occurs throughout the city. While estimates vary, there are approximately 6000 FSWs in Tijuana and 4000 in Ciudad Juarez [19],

Tijuana and Ciudad Juarez are also situated on major drug trafficking routes for heroin, methamphetamine and cocaine, and hence have thriving retail drug markets. ,The number of IDUs in both cities has been estimated at 10,000 and 6,500, respectively [20]. Unlike many countries, syringes can be legally purchased and carried without a prescription, but barriers to purchasing syringes at pharmacies have been reported [21]. In both cities, local non-governmental organizations operate legal NEPs that exchange syringes and provide free condoms and limited STI treatment. High levels of injection and non-injection drug use have also been reported among FSWs and their male clients in Tijuana [22]. These cities are also characterized by high background levels of violence and human rights abuses perpetrated by law enforcement [23].

Despite the potential importance of FSW-IDUs as a subgroup at high risk of acquiring and transmitting HIV/STIs, few studies have empirically assessed their risk factors for HIV infection [24]. Following other epidemiologic and social science researchers [25]–[28], we contend that HIV-related behaviors are shaped by social context and the risk environment. The conceptual framework of risk environment developed by Rhodes and colleagues [6], [25], [26] encourages a focus on interactions between risk factors exogenous to the individual, and posits that physical, social, economic, and political environments interact with microenvironmental and macroenvironmental factors to confer risk or protection for HIV infection. Especially in resource-limited settings, studies are needed to determine whether scarce prevention services should be targeted more towards sexual risks, injection risks or factors in the social, political and economic environment which shape individual-level HIV risk behaviors [6], [25].

We studied FSW-IDUs in Tijuana and Ciudad Juarez to identify correlates of HIV infection in the physical, social economic and policy environment, with the goal of determining whether environmental factors influencing injection risks or sexual risks were most strongly associated with HIV infection. Since an earlier analysis by our team conducted among male IDUs in Tijuana found that policing practices were associated with the risk of HIV infection [29], we were particularly interested to assess whether this held true among FSW-IDUs.

## Methods

Between October, 2008 and October, 2009, FSW-IDUs were recruited into a behavioral intervention study in Tijuana and Ciudad Juarez that aimed to reduce injection and sexual risk behaviors associated with HIV and STI acquisition. Women who appeared to be working as FSWs were unobtrusively approached by outreach workers at bars, street corners and motels to assess study interest and eligibility. Eligibility criteria were: being age ≥18 years; having unprotected vaginal or anal sex with a male client in the previous month; having injected illicit drugs and shared syringes and/or other injection equipment within the past month; ability to speak Spanish or English; being able to provide informed consent; having no plans to permanently move out of the city in the following 18 months and agreeing to accept free STI treatment. At baseline and quarterly thereafter, participants underwent interviewer-administered surveys and biological testing for HIV and STIs. The study protocol was approved by the institutional review boards at UC San Diego, Tijuana General Hospital, and the Universidad Autonoma de Ciudad Juarez. All subjects provided written consent.

### Study Instrument

Participants underwent an interviewer-administered survey eliciting data on sociodemographics, sexual risk behaviors, injection risk behaviors, and experiences reflecting exposures in their physical, social, economic and policy environment in their lifetime and over the last six months. Sociodemographic questions included age, marital status, place of birth, migration history, income and living arrangements. Questions on history of sexual behavior included age at initiation of sex work, reasons for entering sex work. Questions on history of drug use behaviors included age at which they first used injection and non-injection drugs alone or in combination.

Following Rhodes' risk environment framework [6], [26], we considered influences in the physical, social, economic and policy environments, at the micro- and macro-level. Variables in the micro-physical environment include homelessness (i.e., lived mostly on the streets, in an abandoned building or in a car, bus, truck or other vehicle), mobility (i.e., duration of time lived in their current city of residence), number of hours spent on the street, number of lifetime incarcerations and exposure to trauma (sexual and physical abuse experienced as a child and adult, physical and sexual abuse from intimate partners, clients, and police). At the macro-physical level, participants were asked if they had ever crossed from Mexico to the U.S., if they had ever been deported from the U.S., and their perception of the availability and purity of heroin, cocaine and methamphetamine during the prior six months.

Factors at the micro-social environment included both sexual and drug using behaviors occurring in the context of others. Sexual behaviors included frequency of protected and unprotected vaginal and anal sex acts with clients and regular and casual male intimate partners, and frequency of sex with other women. Drug use behaviors included frequency of receptive and distributive sharing of syringes and other injection paraphernalia and the context in which drugs were injected (i.e., where and with whom). We also collected information on how frequently women used drugs and/or alcohol with various types of sex partners. We assessed self-efficacy towards safer injection using a measure that was developed in previous U.S. intervention studies of IDUs [30]. Using a 4-point scale (1 = Strongly Disagree to 4 = Strongly Agree), participants assessed the extent to which they feel they can practice safer injection techniques (e.g., “I can avoid sharing needles even if have had sex without condoms with this person”; “I can avoid sharing needle even if I am in withdrawal (‘*mallila*”). Cronbach's alpha was 0.91. We also assessed self-efficacy towards condom use using a 5-item measure from a previous intervention study of FSWs in Mexico [19], which asks participants to indicate the extent to which they are able to use a condom properly with clients, using the same 4-item response categories as above. Cronbach's alpha was 0.85 [31].

Factors at the micro-policy environment included whether they had ever been tested for HIV or been enrolled in a drug treatment program, and whether they had obtained syringes at a NEP in the last month. We also explored specific policing practices such as being arrested for possessing used or unused/sterile syringes or for having track marks, since these reasons were previously associated with needle sharing and HIV infection among a sample of primarily male IDUs [32], [33]. We further asked whether police had asked them for anything in exchange for not arresting them (e.g., money, sexual favors), and if police had ever confiscated their syringe or solicited sexual favors instead of arresting them in the last six months.

Economic factors at the micro-level included monthly income, household income, amount earned for protected and unprotected sex transactions, how often clients offered more money for sex without a condom and cost of over-the-counter syringes. At the macro-economic level, we asked if participants perceived that the retail purchase price of heroin, cocaine and methamphetamine had increased, decreased or stayed the same over the last six months.

### Laboratory Testing

The “Determine”® rapid HIV antibody test was administered to determine the presence of HIV antibodies (Abbott Pharmaceuticals, Boston, MA). All reactive samples were tested using an HIV-1 enzyme immunoassay and immunofluorescence assay. Those testing HIV-positive were referred to the local municipal health clinics in Tijuana or Cd. Juarez for monitoring and care. Syphilis serology used the rapid plasma reagin (RPR) test (Determine™ Syphilis TP; Abbott Pharmaceuticals, Boston, MA). RPR-positive samples were subjected to confirmatory testing using the *Treponema pallidum* particle agglutination assay (TPPA) (Fujirebio, Wilmington, DE, USA). Women testing positive were treated presumptively with benzicillin injections (once per week, for three weeks).

Initially, Gonorrhea and Chlamydia were detected using a rapid test kit (BioStar® OIA® GC and CHLAMYDIA) and positive samples were confirmed on urine specimens using TMA (Genprobe, San Diego, CA). Women with positive rapid tests for either infection, or women with STI symptoms were treated presumptively. However, upon release of a report from the US Centers for Disease Control and Prevention that questioned the sensitivity of the BioStar rapid GC test [34], this test was discontinued on March 24, 2009. After this date, all participants provided urine for GC screening using the Genprobe Transcription-Mediated Amplification assay (TMA; San Diego, CA).

Trichomonas was detected using the OSOM® Trichomonas Rapid Test, and Bacterial Vaginosis using the OSOM® BVBlue® Test (Genzyme diagnostics, San Diego, CA). Samples were batched regularly and shipped to the San Diego County Health Department where confirmatory tests were conducted.

### Statistical Analysis

Statistical analyses were conducted on baseline data, comparing HIV-positive and HIV-negative participants, using t-tests and Wilcoxon's Rank Sum tests for continuous normally and non-normally distributed variables, respectively. Binary outcomes were examined using Pearson's Chi-square or Fisher's exact tests. Univariate and multivariate logistic regressions were performed to identify factors associated with HIV-positive serostatus, considering sociodemographics and aforementioned factors at the micro- and macro- level of the risk environment. A manual procedure was used whereby all variables attaining significance levels <10% in univariate models were considered for inclusion in multivariate models. Although not significant in univariate analyses, we also considered receptive syringe sharing in multivariate models since it is a known HIV risk factor. The likelihood ratio test was used to compare nested models, using a significance level of 5%. Plausible interactions were explored.

## Results

Of 620 FSW-IDUs (309 from Tijuana, 311 from Ciudad Juarez), median age and duration in sex work were 33 (interquartile range [IQR]: 27–40) and 11 years (IQR: 6–17), respectively. Of these 620 women, 33 (5.3%) tested HIV-positive, of whom 27 (82%) were previously unaware of their infection. Prevalence of gonorrhea, Chlamydia, trichomonas, bacterial vaginosis, syphilis titers ≥1∶8, or any STI (including HIV) was 4%, 13%, 35%, 39%, 10% and 72%, respectively. After changes in testing for gonorrhea were implemented, we observed no significant increase in the positivity rate (results not shown). As seen in Table 1 and 2, compared to other women, HIV-positive women were more likely to have lifetime syphilis infection (58% vs. 26%, p<0.001) and syphilis titers ≥1∶8 (36% vs. 9%, p<0.001). HIV-positive women were also more likely to test positive for *Trichomonas* (58% vs. 34%, p = 0.01), but were no more likely to test positive for gonorrhea or Chlamydia.

**Table 1:** Sociodemographic, Biologic and Behavioral Characteristics of FSW-IDUs with and without HIV Infection in Tijuana and Ciudad Juarez.

Baseline Characteristics	HIV+ (n = 33)	HIV-neg (n = 587)	Total (n620)	P	Odds Ratio (95%CI)
**Sociodemographics**					
Age*	31 (28–35)	33 (27–40)	33 (27–40)	0.17	0.97 (0.93–1.01)
Education completed*	6 (4–8)	6 (5–9)	6 (5–9)	0.2	0.92 (0.83–1.03)
Speaks English	4 (12%)	160 (27%)	164 (26%)	0.07	0.37 (0.13–1.06)
Married/common law	15 (45%)	219 (37%)	234 (38%)	0.36	1.4 (0.69–2.84)
**Biologic Factors**					
Gonorrhea	2 (6%)	20 (3%)	22(4%)	0.34	1.79 (0.40–8.02)
Chlamydia	8 (24%)	72 (12%)	80 (13%)	0.06	2.28 (0.99–5.25)
Syphilis titer ≥1∶8	12 (36%)	52 (9%)	64 (10%)	<.001	5.88 (2.74–12.63)
Any syphilis	19 (58%)	155 (26%)	174 (28%)	<.001	3.78 (1.85–7.73)
Trichomonas	19 (58%)	199 (34%)	218 (35%)	0.01	2.65 (1.30–5.39)
Bacterial Vaginosis	14 (42%)	228 (39%)	242 (39%)	0.72	1.16 (0.57–2.36)
**Lifetime Individual Risk Behaviors**					
Age when first traded sex*	19 (17–21)	19 (15–23)	19 (15–23)	0.55	1 (0.95–1.06)
Years spent as a sex worker*	10 (7–15)	11 (6–17)	11 (6–17)	0.3	0.97 (0.92–1.01)
Age at first injection*	19 (16–25)	20 (17–26)	20 (17–26)	0.33	0.97 (0.92–1.03)
**Individual Current Risk Behaviors**					
Any receptive needle sharing†	31 (94%)	559(95%)	590 (95%)	0.66	0.75 (0.17–3.29)
Shared injection paraphernalia ≥half the time†	11 (33%)	293 (50%)	304 (49%)	0.07	0.5 (0.24–1.05)
No. male clients (per 10 clients)†	1.6 (1–6)	3 (1–8)	3 (1–8)	0.08	0.97 (0.90–1.05)

*Median (years);

† Past month.

NOTE: Certain percentages may reflect denominators smaller than the N value given in the column head. These discrepancies are due to missing data.

**Table 2:** Environmental Factors Associated With HIV Infection among FSW-IDUs in Tijuana and Ciudad Juarez.

Baseline Characteristics	HIV+ (n = 33)	HIV−(n = 587)	Total (n = 620)	P	Odds Ratio (95%CI)
**Physical Risk Environment**					
*Micro-physical*					
Lived in Tijuana/Cd. Juarez whole life	16 (48%)	256 (44%)	272 (44%)	0.59	1.22(0.60–2.46)
Median # hours spent on street (IQR)*	12 (8–16)	10 (7–15)	10 (7–15)	0.55	1.01 (0.95–1.07)
Mostly homeless†	0 (0%)	35 (6%)	35 (6%)	0.25	4.1 (0.26–65.5) (0.26666566565.50)
Sexually abused as a child	9 (28%)	196 (34%)	205 (33%)	0.57	0.77 (0.35–1.69)
Physically abused as a child	7 (22%)	143 (25%)	150 (25%)	0.83	0.85 (0.36–2.02)
Sexual abused/raped by client*	8 (24%)	130 (23%)	138 (23%)	0.83	1.1 (0.48–2.49)
Sexual abused/raped by intimate partner*	2 (6%)	32 (6%)	34 (6%)	0.7	1.13 (0.26–4.95)
Have been incarcerated	27 (82%)	431 (73%)	458 (74%)	0.41	1.63 (0.66–4.02)
*Macro-physical*					
Ever traveled to United States	17 (52%)	301 (51%)	318 (51%)	1	1.01 (0.50–2.04)
Ever deported from United States	3 (9%)	52 (9%)	55 (9%)	1	1.03 (0.30–3.49)
**Social Risk Environment**					
*Micro-social*					
Median # people usually injected with†(IQR)	2 (1–4)	3 (1–5)	3 (1–5)	0.18	0.96 (0.89–1.05)
Infrequent condom use with clients during vaginal sex†	6 (19%)	172 (30%)	178 (30%)	0.23	0.54 (0.22–1.32)
Infrequent condom use with clients during anal sex†	1 (10%)	55 (27%)	56 (26%)	0.46	0.31 (0.04–2.48)
Injected drugs with a client often/always†	18 (55%)	186 (32%)	204 (33%)	0.01	2.58 (1.27–5.23)
Injected drugs with intimate sex partner/spouse/family†	10 (30%)	122 (21%)	132 (21%)	0.2	1.64 (0.76–3.54)
Self efficacy score for needle sharing (IQR)	2.2 (2–2.8)	2 (1.8–2.7)	2 (1.8–2.7)	0.06	1.67 (0.95–2.91)
Self-efficacy score for condom use (IQR)	3 (2.4–3)	3 (2.6–3)	3 (2.6–3)	0.65	0.94 (0.50–1.78)
**Economic Risk Environment**					
*Micro-economic*					
Average monthly income ≥3500 pesos	18 (55%)	276 (47%)	294 (47%)	0.47	1.35 (0.67–2.73)
Median amount earned per unprotected sex transaction**	18.8 (12–30)	20 (15–30)	20 (15–30)	0.18	0.97 (0.94–1.01)
Median amount earned per protected sex transaction**	15 (10–22.5)	17.5 (10–25)	17.5 (10–25)	0.41	0.98 (0.95–1.02)
Reported earning more for unprotected sex*	14 (50%)	270 (47%)	284 (47%)	0.85	1.11 (0.52–2.37)
Police solicited bribes instead of arresting them*	24 (73%)	365 (62%)	389 (63%)	0.27	1.61 (0.73–3.52)
**Policy Risk Environment**					
*Micro-policy*					
Ever tested for HIV	23 (70%)	300 (51%)	323 (52%)	0.05	2.19 (1.03–4.69)
Attended needle exchange program†	8 (24%)	60 (10%)	68 (11%)	0.02	2.81 (1.21–6.50)
Ever enrolled in drug treatment	19 (58%)	300 (51%)	319 (52%)	0.59	1.29 (0.64–2.63)
Police confiscated syringes instead of arresting them*	16 (48%)	166 (28%)	182 (29%)	0.02	2.38 (1.17–4.81)

IQR = inter-quartile range.

*Past 6 months;

† Past month. NOTE: Certain percentages may reflect denominators smaller than the N value given in the column head. These discrepancies are due to missing data.

**USD.

Baseline comparisons of HIV-positive and HIV-negative women suggested that the two groups were similar with respect to sociodemographics (Table 1); groups did not differ significantly in age, education, language spoken, or marital status. Similarly, groups did not differ in their lifetime individual risk behaviors: that is, there were no significant differences in age when women first traded sex, the number of years they reported being in sex work, nor when they first injected drugs. Similarly, current risk behaviors did not differ: there were no significant differences between HIV-positive and HIV-negative women in receptive needle sharing, sharing injection paraphernalia or the number of male clients.

We next compared HIV-positive and HIV-negative women according to exposures in their physical, social and policy risk environments. As shown in Table 2, groups did not differ in regards to their micro-physical environment (i.e., living in Tijuana/Cd. Juarez for their entire life, time spent on the street, homelessness, childhood sexual or physical abuse, having been raped or sexually abused by either a client or intimate partner, or having been incarcerated). Nor did the two groups differ in terms of influences in the macro-physical environment (i.e., ever traveled to the United States, ever deported from the United States).

In terms of their social risk environment, the only significant difference was that compared to HIV-negative women, HIV-positive women were significantly more likely to report often or always injecting drugs with clients (Odds ratio [OR]: 2.58). The two groups did not differ in reports of injecting drugs with their intimate sex partner, spouse or family member, nor did they differ significantly according to the number of people they usually injected with. Finally, similar percentages of HIV-positive and HIV-negative women reported infrequently using condoms for vaginal (30% of the time) and anal (26% of the time) sex with clients.

HIV-positive and HIV-negative women did not differ according to influences in their economic risk environment. No differences were reported for average monthly income, amount earned for unprotected or protected sex, or reporting that the police solicited bribes instead of arresting them. However it is noteworthy that the large majority (63%) reported that police solicited bribes in lieu of arresting them in the last six months. Almost half (47%) reported earning more for unprotected sex than for protected sex.

In assessing the micro-policy risk environment, compared to HIV-negative women, HIV-positive women were significantly more likely to have ever been tested for HIV (70% vs. 51%, p = 0.05), and were more likely to report that the police confiscated their syringes instead of arresting them (48% vs. 28%, p = 0.02). HIV-positive women were more likely to have obtained syringes at NEPs within the last month (24% vs. 10%, p = 0.02). Approximately half of women reported ever (or currently) being enrolled in a drug treatment program, but there were no differences between the two groups.

**Figure 1:**
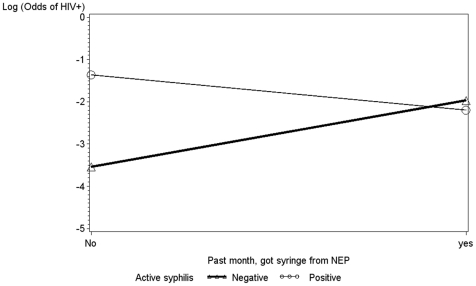
Odds of HIV infection for IDUs with (○ ○ ○) and without (Δ Δ Δ) active syphilis by needle exchange attendance.

In a final multivariate regression model (Table 3), factors independently associated with HIV infection were syphilis titers ≥1∶8, often/always injecting drugs with clients, police confiscation of syringes in lieu of arrest, and obtaining syringes from NEPs within the last month. We explored a possible interaction between police confiscation of syringes and NEP attendance on the odds of HIV infection, since IDUs had previously raised concerns that police would harass them at NEPs [21]. This interaction was not significant. We also examined a possible interaction between syphilis infection and NEP attendance on the odds of HIV infection, since the local NGOs operating needle exchange also offer condoms and limited on-site STI treatment. We observed a significant interaction indicating that for women who did not obtain syringes from NEPs within the last month, the odds of HIV infection associated with syphilis infection was significantly elevated (Figure 1). On the other hand, for women who obtained syringes from the NEP within the last month, there was no significant association between active syphilis infection and the odds of HIV infection.

**Table 3:** Factors Independently Associated With HIV Infection among FSW-IDUs in Tijuana and Ciudad Juarez.

Variable	Adjusted OR	95% CI
Injected drugs with a client often/always**	3.02	1.40–6.52
Police confiscated syringe in exchange for not arresting them*	2.4	1.16–4.99
Syphilis titers ≥1∶8	10.15	4.14–24.88
Obtained syringes at needle exchange program**	5.43	2.02–14.58
Attended needle exchange×syphilis titers ≥1∶8	0.08	0.01–0.70

*Past 6 months.

**last month.

## Discussion

This large study of HIV risks among FSW-IDUs living in two Mexico-US border cities uncovered two important findings. First, beyond known biological HIV risk factors such as infectious syphilis, factors operating in both the micro-social environment (i.e., injecting drugs with clients) and policy environment (i.e., having syringes confiscated by police, attending NEPs) predominated as factors associated with risk of HIV infection, rather than individual-level risk behaviors. Second, factors associated with injection behaviors were more closely associated with HIV infection than sexual behaviors. These findings have salient implications for structural interventions that are needed to interrupt HIV transmission behaviors among FSWs who inject drugs.

Over half of HIV-positive FSW-IDUs in these cities reported injecting drugs with clients, which presumably occurred in the context of a sexual transaction. These data are supported by a recent study of male clients of FSWs in Tijuana, where HIV prevalence was 5% and nearly half reported that they were high ‘very often’ or ‘fairly often’ when having sex with an FSW [22]. In our study, injecting with clients was associated with three-fold higher odds of HIV infection, which could be a marker of HIV transmission occurring through shared injection equipment or unprotected sex. One possible interpretation for the elevated HIV risk we observed is that sex workers who are dependent on clients for their drug supply may have lower self-efficacy for negotiating condom use, a finding that is supported by a large study of non-commercial partnerships [35]. However, self-efficacy scores for condom use and refusing needle sharing were not independently associated with HIV infection. A second interpretation is that these women are exposed to high levels of violence, exacerbated by their own and/or their client's drug use, leading them to acquiesce to clients' demands for sex without a condom [14], [36].

Regardless of the mechanism, addiction appears to be a major driver of HIV risks among FSW-IDUs, highlighting the need for greater access to drug treatment for this population, which is also a priority for Mexican policymakers [37]. Behavioral interventions should also focus on reducing high risk behaviors among male clients, who should acknowledge their shared responsibility in protecting themselves and their sex partners from HIV/STIs. From a harm reduction perspective, prevention programs should encourage FSWs and clients to try to delay drug use until after sexual transactions are complete to ensure that their ability to negotiate condom use is not unduly compromised.

Our study also found that police confiscation of syringes was independently associated with more than two-fold higher odds of HIV infection. Previous studies in Tijuana and Ciudad Juarez found that drug users who had been arrested for carrying sterile or used syringes were three times more likely to report receptive needle sharing [32] and twice as likely to attend shooting galleries [38]. Among male IDUs, being arrested for having “track marks” was independently associated with HIV infection in Tijuana [29], suggesting that multiple policing practices may be linked with elevated risk of HIV transmission. Although our cross-sectional analysis precludes drawing causal inferences, the fact that HIV-positive women are more likely to have their syringes confiscated can increase their likelihood of syringe sharing or experiencing gender-based violence [39], both which may fuel HIV transmission. It is also possible, however, that some uncontrolled for factors contribute both to increased risk for encounters with police and HIV risk behavior among IDUs. Despite the growing body of evidence drawing links between IDUs' and FSWs' experience of police abuse and HIV risk, concrete psychological, ecological, or other pathways underlying these associations have not been quantitatively assessed. Robust multi-level research using both qualitative and quantitative methods is crucial to inform educational, policy, and other structural interventions designed to harmonize law enforcement with public health goals and programs. Police education programs designed to align policing with public health efforts targeting vulnerable populations have shown promise in other settings, especially when information about public health programs and policies is bundled with occupational safety content of concern to police (e.g., prevention of needle-stick injuries) [40], [41].

The strong association between HIV infection and syphilis titers consistent with active infection was anticipated, since the latter is a well established cofactor of HIV transmission [42] and because this association was previously reported among IDUs, FSWs and their clients in Tijuana [19], [22], [29]. However, the observation that NEP attendance modified the association between odds of HIV infection and active syphilis infection was unexpected. The most obvious explanation is that FSWs obtaining syringes at NEPs also obtain condoms which are available at these programs (thereby lowering their risk of acquiring syphilis), or obtain STI treatment there (thereby lowering their syphilis titers). Another possible explanation is that by obtaining syringes from NEPs, FSW-IDUs are at lower risk of acquiring syphilis parenterally through needle sharing. An earlier report from our team suggested that parenterally syphilis infection was occurring through shared injection equipment in Tijuana and Ciudad Juarez [43], following a similar report from Russia [44]. Prospective studies are required to disentangle these associations, but both explanations support the important role of NEPs in providing IDUs with syringes and other ancillary HIV/STI prevention and treatment services. Such efforts will need the ongoing support of Mexican authorities at the municipal, state and federal levels and appropriate resource allocation through sources such as the Global Fund to Prevent HIV, TB and Malaria.

Our study was limited by a number of factors, including a relatively low HIV prevalence and initial use of a rapid test for Gonorrhea which had low sensitivity [34], possibly masking some associations. Since these baseline data were collected from a behavioral intervention study designed to lower both risky sexual and injection behaviors, we selected FSW-IDUs who had engaged in both recent unprotected sex and sharing of injection equipment who are unlikely to be representative of most FSWs in these cities. However, the finding that two-thirds of FSW-IDUs had at least one current STI is sobering, and suggests that rapid escalation of HIV infection could ensue.

In summary, structural factors predominated as drivers of HIV infection among FSW-IDUs in Tijuana and Ciudad Juarez, extending earlier findings among male and female IDUs [29], [32]. Interventions to prevent, monitor and address unjustified police practices, reduce risky behaviors among clients, and offer ancillary HIV/STI prevention services through NEPs should be pursued as strategies to reduce HIV risk in the Mexico-US border region. Researchers should consider different levels and types of the HIV risk environment that shape risk behaviors in vulnerable populations at high risk for HIV infection, thereby shifting the onus of responsibility for behavior change away from individuals and towards actors in the policy arena.
